# Supplementary material for: A combined adjuvant approach primes robust germinal center responses and humoral immunity in non-human primates
Source: Nat Commun. 2023 Nov 4;14:7107. doi: 10.1038/s41467-023-42923-x (PMC10625619; doi:10.1038/s41467-023-42923-x)
Supplement: Supplementary file 1 — Supplementary Information [file 41467_2023_42923_MOESM1_ESM.pdf]

A combined adjuvant approach primes robust germinal center responses and humoral immunity in non-human primates

Ivy Phung[1,2,3], Kristen A. Rodrigues[4,5], Ester Marina Zarate[1,2], Laura Maiorino[4,5], Bapi Pahar[6], Wen-Hsin Lee[7], Mariane Melo[2,4,5], Amitinder Kaur[6], Carolina Allers[6], Marissa Fahlberg[6], Brooke F. Grasperge[6], Jason P. Dufour[6], Faith Schiro[6], Pyone P. Aye[6], Paul G. Lopez[1], Jonathan L. Torres[7], Gabriel Ozorowski[2,7,8], Saman Eskandarzadeh[2,8,9], Michael Kubitz[2,8,9], Erik Georgeson[2,8,9], Bettina Groschel[2,8,9], Rebecca Nedellec[9], Michael Bick[9], Katarzyna Kaczmarek Michaels[4,5], Hongmei Gao[10], Xiaoying Shen[10], Diane G. Carnathan[11], Guido Silvestri[11], David C. Montefiori[10], Andrew B. Ward[2,7], Lars Hangartner[2,9], Ronald S. Veazey[6], Dennis R. Burton[2,5,8,9], William R. Schief[2,5,8,9], Darrell J. Irvine[2,4,5,12,13,14*], Shane Crotty[1,2,3,*]

[1]Center for Infectious Disease and Vaccine Research, La Jolla Institute for Immunology (LJI), La Jolla, CA 92037, USA. [2]Consortium for HIV/AIDS Vaccine Development (CHAVD), The Scripps Research Institute, La Jolla, CA 92037 USA. [3]Department of Medicine, Division of Infectious Diseases and Global Public Health, University of California, San Diego (UCSD), La Jolla, CA 92037, USA.[4]Koch Institute for Integrative Cancer Research, Massachusetts Institute of Technology, Cambridge, MA 02139 USA. [5]Ragon Institute of Massachusetts General Hospital, Massachusetts Institute of Technology and Harvard University, Cambridge, MA 02139 USA. [6]Tulane National Primate Research Center, Tulane School of Medicine, Covington, LA 70433 USA.[7]Department of Integrative Structural and Computational Biology, The Scripps Research Institute, La Jolla, CA 92037 USA.[8]IAVI Neutralizing Antibody Center, The Scripps Research Institute, La Jolla, CA 92037 USA.[9]Department of Immunology and Microbiology, The Scripps Research Institute, La Jolla, CA 92037 USA. [10]Department of Surgery, Laboratory for AIDS Vaccine Research & Development, Duke University Medical Center, Duke University, Durham, NC 27710 USA. [11]Emory National Primate Research Center and Emory Vaccine Center, Emory University School of Medicine, Atlanta, GA 30322 USA. [12]Department of Biological Engineering, Massachusetts Institute of Technology, Cambridge, MA 02139 USA. [13]Department of Materials Science and Engineering, Massachusetts Institute of Technology, Cambridge, MA 02139 USA. [14]Howard Hughes Medical Institute, Chevy Chase, MD 20815 USA. *email: djirvine@mit.edu; shane@lji.org

Supplementary Figures 1-8

Supplementary Tables 1-3

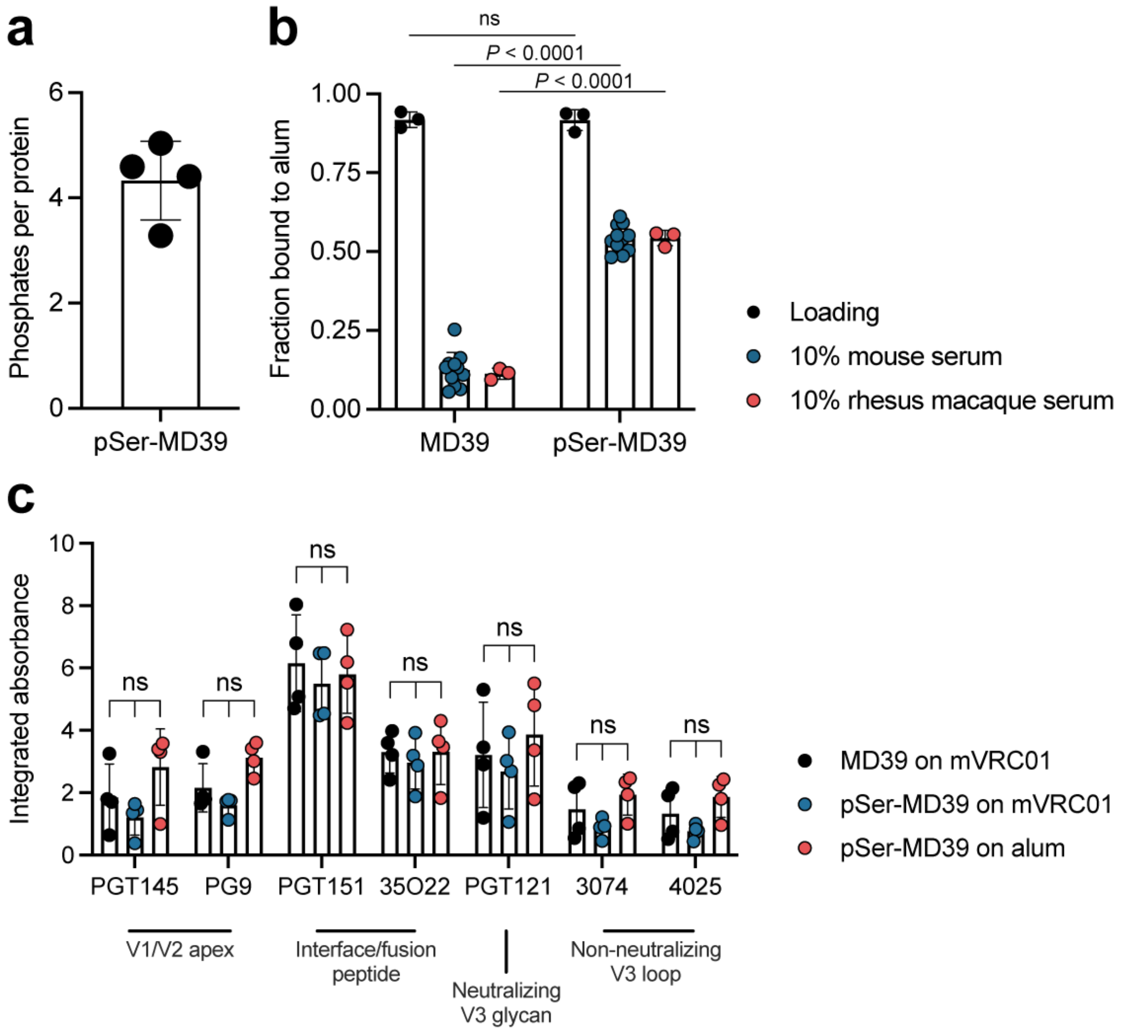

Supplementary Fig. 1 | pSer-conjugated MD39 exhibits strong alum binding and retains the antigenicity profile of MD39.

a, MD39 conjugated to peptides containing 4 phosphoserines (pSer-MD39) was assayed for phosphates by a malachite green assay. **b,** pSer-conjugated or unmodified MD39 trimers were mixed with alum, and the fraction of protein bound to alum was assessed by after initial 30-minute adsorption ("Loading") or after 24-hour incubation in mouse or rhesus macaque serum at 37°C. **c,** Antigenicity profiling of unmodified MD39 captured by mouse VRC01 compared to pSer-MD39 on alum or captured by mouse VRC01. Shown are area-under-the-curve values for trimer binding vs. antibody concentration. Mean and SD are plotted. Statistical significance was tested using two-way ANOVA with Sidak's multiple comparisons test in (b) or Tukey's multiple comparisons test in (c). p < 0.05, **p < 0.01, ***p < 0.001, ****p < 0.0001. Source data are provided as a Source Data file.

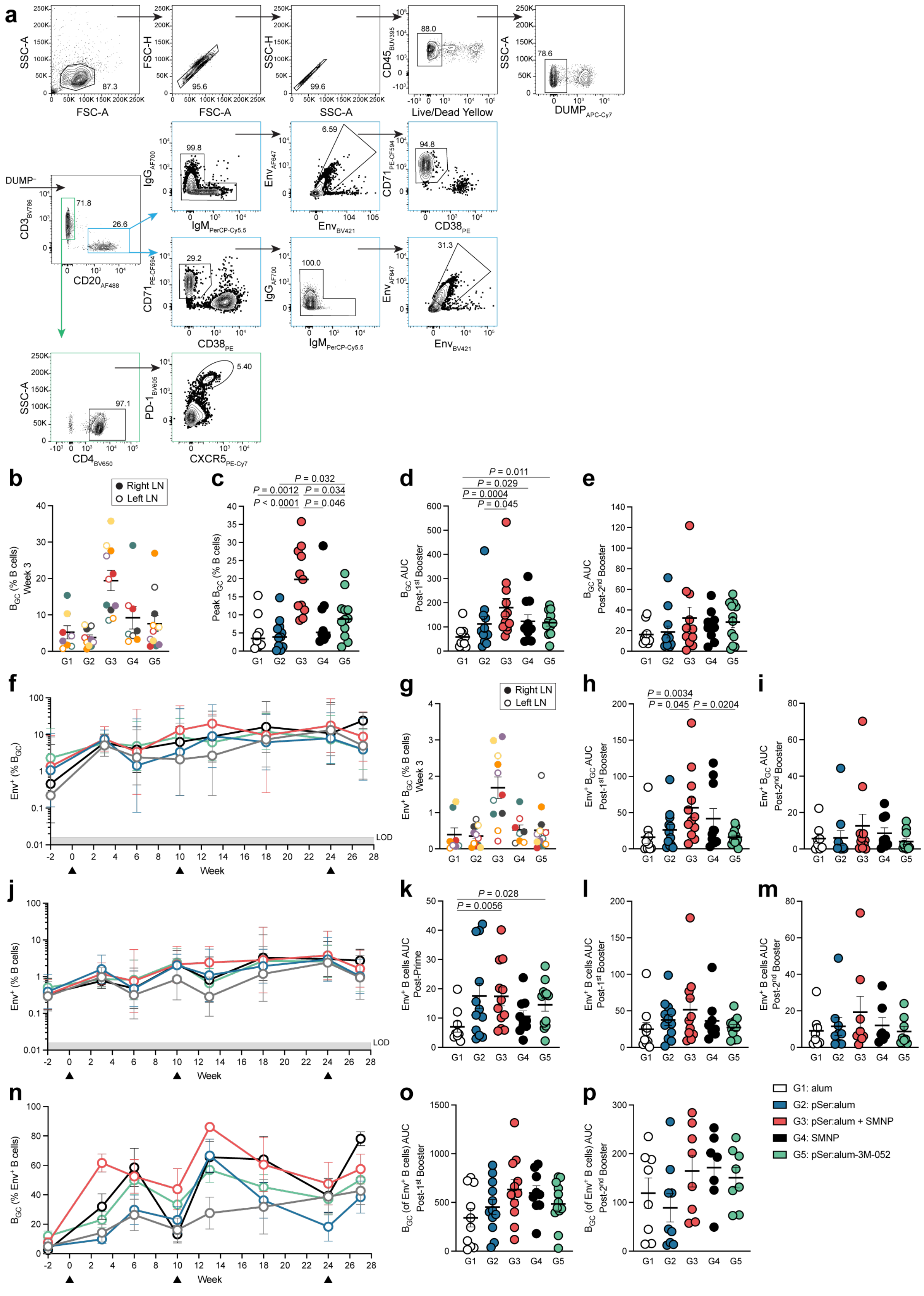

a
SSC-A
FSC-A
87.3
FSC-H
95.6
SSC-H
SSC-A
99.6
CD45BUV395
Live/Dead Yellow
88.0
DUMPAPC-Cy7
78.6
DUMP⁻
CD3BV786
CD20AF488
71.8
26.6
IgGAF700
IgMPerCP-Cy5.5
99.8
EnvAF647
EnvBV421
6.59
CD71PE-CF594
CD38PE
94.8
29.2
100.0
31.3
CD4BV650
97.1
PD-1BV605
CXCR5PE-Cy7
5.40
b
Right LN
Left LN
BGC (% B cells) Week 3
c
Peak BGC (% B cells)
P = 0.032
P = 0.0012
P = 0.034
P < 0.0001
P = 0.046
d
BGC AUC Post-1st Booster
P = 0.011
P = 0.029
P = 0.0004
P = 0.045
e
BGC AUC Post-2nd Booster
f
Env+ (% BGC)
LOD
Week
g
Env+ BGC (% B cells) Week 3
Right LN
Left LN
h
Env+ BGC AUC Post-1st Booster
P = 0.0034
P = 0.045
P = 0.0204
i
Env+ BGC AUC Post-2nd Booster
j
Env+ (% B cells)
LOD
Week
k
Env+ B cells AUC Post-Prime
P = 0.028
P = 0.0056
l
Env+ B cells AUC Post-1st Booster
m
Env+ B cells AUC Post-2nd Booster
n
BGC (% Env+ B cells)
Week
o
BGC (of Env+ B cells) AUC Post-1st Booster
p
BGC (of Env+ B cells) AUC Post-2nd Booster
G1: alum
G2: pSer:alum
G3: pSer:alum + SMNP
G4: SMNP
G5: pSer:alum-3M-052

Supplementary Fig. 2 | B_{GC} and Env-specific B cell kinetics.

a, Gating strategy for analysis of B_{GC} cell dynamics. **b,** B_{GC} cell frequency as a percentage of B cells at week 3 comparing left and right draining lymph node (LN) frequencies. The filled circles indicate the right LN and the empty circles indicate the left LN. The color of the circles distinguishes each animal within the immunization groups and also to identify left-right LN pairs. **c,** Peak B_{GC} cell frequencies as a percentage of B cells after priming immunization. **d,** AUC of B_{GC} cell frequency post-1st booster immunization. **e,** AUC of B_{GC} cell frequency post-2nd booster immunization. **f,** Env-binding frequency as a percentage of B_{GC} cells. **g,** Env-binding B_{GC} cells as a percentage of B cells at week 3, comparing left and right draining LN frequencies. **h,** AUC of Env-binding B_{GC} cell frequency post-1st booster immunization. **i,** AUC of Env-binding B_{GC} cell frequency post-2nd booster immunization. **j,** Env-binding frequency as a percentage of B cells. **k,** AUC of Env-binding B cell frequency post-priming immunization. **l,** AUC of Env-binding B cell frequency post-1st booster immunization. **m,** AUC of Env-binding B cell frequency post-2nd booster immunization. **n,** Longitudinal analysis of the proportion of Env-binding B cells that were B_{GC} cells. **o,** AUC of Env-binding B cells that were B_{GC} post-1st booster immunization. **p,** AUC of Env-binding B cells that were B_{GC} post-2nd booster immunization. Black triangles represent time of immunization. Mean and SEM or geometric mean and geometric SD are plotted depending on the scale in all figures unless otherwise stated. For Supplementary Fig. 2, n=12 samples (left and right LN), 6 animals per group. Statistical significance was tested using either unpaired two-tailed Mann-Whitney tests or Kruskal-Wallis test with Dunn's multiple comparisons test, depending on the objectives of the study. $^{*}p < 0.05$, $^{**}p < 0.01$, $^{***}p < 0.001$, $^{****}p < 0.0001$. Source data are provided as a Source Data file.

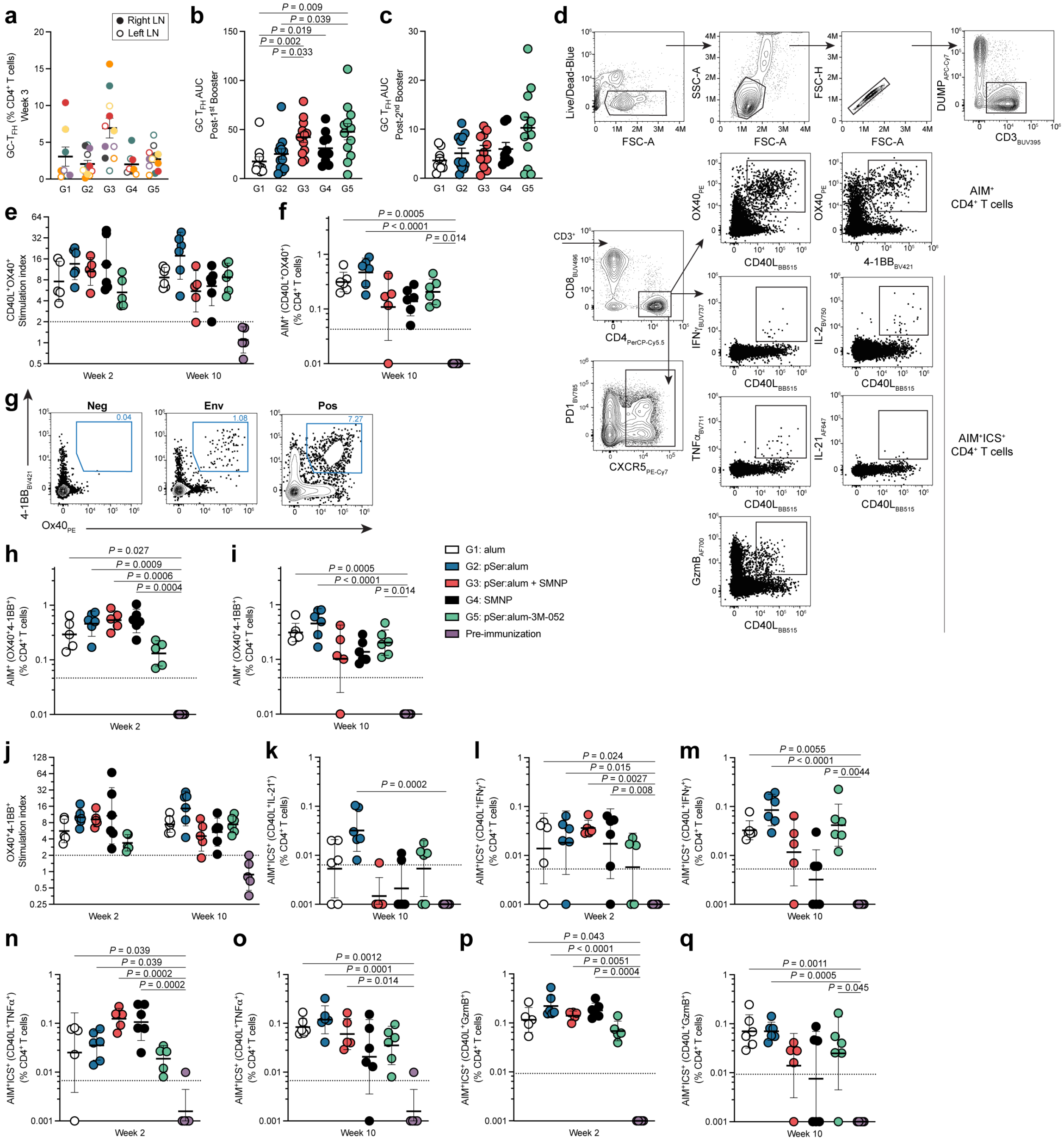

a
Right LN
Left LN
GC-TFH (% CD4+ T cells) Week 3
G1 G2 G3 G4 G5
b
P = 0.009
P = 0.039
P = 0.019
P = 0.002
P = 0.033
GC TFH AUC Post-1st Booster
G1 G2 G3 G4 G5
c
GC TFH AUC Post-2nd Booster
G1 G2 G3 G4 G5
d
Live/Dead-Blue
FSC-A
SSC-A
FSC-A
FSC-H
FSC-A
DUMP APC-Cy7
CD3 BUV395
CD3+
CD8 BUV496
CD4 PerCP-Cy5.5
PD1 BV785
CXCR5 PE-Cy7
OX40 PE
CD40L BB515
OX40 PE
4-1BB BV421
AIM+ CD4+ T cells
IFNγ BUV737
CD40L BB515
IL-2 BV750
CD40L BB515
TNFα BV711
CD40L BB515
IL-21 AF647
CD40L BB515
GzmB AF700
CD40L BB515
AIM+ICS+ CD4+ T cells
e
CD40L+OX40+ Stimulation index
Week 2
Week 10
f
P = 0.0005
P < 0.0001
P = 0.014
AIM+ (CD40L+OX40+) (% CD4+ T cells)
Week 10
g
Neg
Env
Pos
0.04
1.08
7.27
4-1BB BV421
Ox40 PE
h
P = 0.027
P = 0.0009
P = 0.0006
P = 0.0004
AIM+ (OX40+4-1BB+) (% CD4+ T cells)
Week 2
i
P = 0.0005
P < 0.0001
P = 0.014
AIM+ (OX40+4-1BB+) (% CD4+ T cells)
Week 10
G1: alum
G2: pSer:alum
G3: pSer:alum + SMNP
G4: SMNP
G5: pSer:alum-3M-052
Pre-immunization
j
OX40+4-1BB+ Stimulation index
Week 2
Week 10
k
P = 0.0002
AIM+ICS+ (CD40L+IL-21+) (% CD4+ T cells)
Week 10
l
P = 0.024
P = 0.015
P = 0.0027
P = 0.008
AIM+ICS+ (CD40L+IFNγ+) (% CD4+ T cells)
Week 2
m
P = 0.0055
P < 0.0001
P = 0.0044
AIM+ICS+ (CD40L+IFNγ+) (% CD4+ T cells)
Week 10
n
P = 0.039
P = 0.039
P = 0.0002
P = 0.0002
AIM+ICS+ (CD40L+TNFα+) (% CD4+ T cells)
Week 2
o
P = 0.0012
P = 0.0001
P = 0.014
AIM+ICS+ (CD40L+TNFα+) (% CD4+ T cells)
Week 10
p
P = 0.043
P < 0.0001
P = 0.0051
P = 0.0004
AIM+ICS+ (CD40L+GzmB+) (% CD4+ T cells)
Week 2
q
P = 0.0011
P = 0.0005
P = 0.045
AIM+ICS+ (CD40L+GzmB+) (% CD4+ T cells)
Week 10

Supplementary Fig. 3 | GC-T_{FH} kinetics and Env-specific T cell responses.
a, GC-T_{FH} cell frequency as a percentage of CD4+ T cells, comparing left and right draining LNs. The filled circles indicate right LN and the empty circles indicate left LN. The color of the circles distinguishes each animal within the immunization groups and also to identify left-right LN pairs. **b,** AUC of GC-T_{FH} cell frequency post-1st booster immunization. **c,** AUC of GC-T_{FH} cell frequency post-2nd booster immunization. **d,** Flow cytometry gating strategy of AIM assay to detect Env-specific T cells and of intracellular cytokine staining assay (ICS) to assess cytokine function. **e,** Stimulation index for $CD40L^+OX40^+$ $CD4^+$ T cells at week 2 and week 10. **f,** AIM^+ ($CD40L^+OX40^+$) T cell frequency as a percentage of CD4+ T cells at week 10. **g,** Representative flow plots of AIM^+ ($OX40^+4\text{-}1BB^+$) $CD4^+$ T cells. **h,** AIM^+ ($OX40^+4\text{-}1BB^+$) T cell frequencies as a percentage of $CD4^+$ T cells at week 2. **i,** AIM^+ ($OX40^+4\text{-}1BB^+$) T cell frequencies as a percentage of $CD4^+$ T cells at week 10. **j,** Stimulation index for $OX40^+4\text{-}1BB^+$ $CD4^+$ T cells at week 2 and week 10. **k,** AIM^+ICS^+ ($CD40L^+IL\text{-}21^+$) of $CD4^+$ T cells at week 10. **l,** AIM^+ICS^+ ($CD40L^+IFN\gamma^+$) of $CD4^+$ T cells at week 2. **m,** AIM^+ICS^+ ($CD40L^+IFN\gamma^+$) of $CD4^+$ T cells at week 10. **n,** AIM^+ICS^+ ($CD40L^+TNF\alpha^+$) of $CD4^+$ T cells at week 2. **o,** AIM^+ICS^+ ($CD40L^+TNF\alpha^+$) of $CD4^+$ T cells at week 10. **p,** AIM^+ICS^+ ($CD40L^+GzmB^+$) of $CD4^+$ T cells at week 2. **q,** AIM^+ICS^+ ($CD40L^+GzmB^+$) of $CD4^+$ T cells at week 10. Black dotted lines indicate the limit of quantification. Black triangles represent time of immunization. Mean and SEM or geometric mean and geometric SD are plotted depending on the scale in all figures unless otherwise stated. For Supplementary Fig. 3a-c, n=12 samples (left and right LN), 6 animals per group. For Supplementary Fig. 3d-q, n=6 samples per group. Statistical significance for GC-T_{FH} cell frequency (a-b) was tested using unpaired two-tailed Mann-Whitney test or Kruskal-Wallis test with Dunn's multiple comparisons test, depending on the objectives of the study. Statistical significance for Env-specific AIM assays (c-p) were tested using Kruskal-Wallis test with uncorrected Dunn's multiple comparisons test. *$p < 0.05$, **$p < 0.01$, ***$p < 0.001$, ****$p < 0.0001$. Source data are provided as a Source Data file.

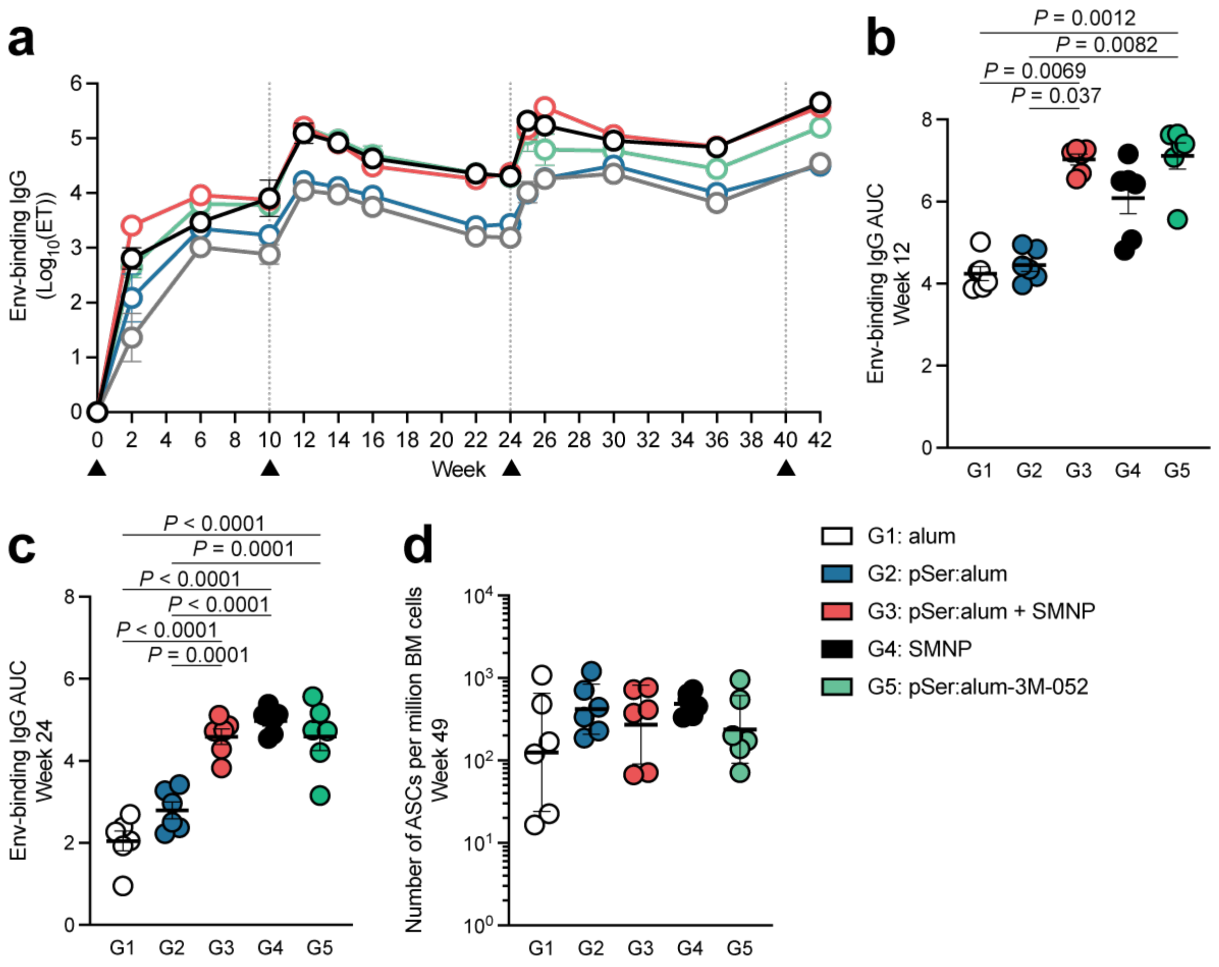

Supplementary Fig. 4 | Env-binding IgG antibody kinetics.
a, Full time course of the Env-binding IgG endpoint titers, measured by ELISA. **b,** AUC of Env-binding serum IgG at week 12. **c,** AUC of Env-binding serum IgG at week 24. **d,** Total IgG ELISpot performed at week 49. Black triangles represent time of immunization. Mean and SEM or geometric mean and geometric SD are plotted depending on the scale in all figures unless otherwise stated. For Supplementary Fig. 4, n=6 samples per group. Statistical significance was tested using either unpaired two-tailed Mann-Whitney tests or Kruskal-Wallis test with Dunn's multiple comparisons test, depending on the objectives of the study. *p < 0.05, **p < 0.01, ***p < 0.001, ****p < 0.0001. Source data are provided as a Source Data file.

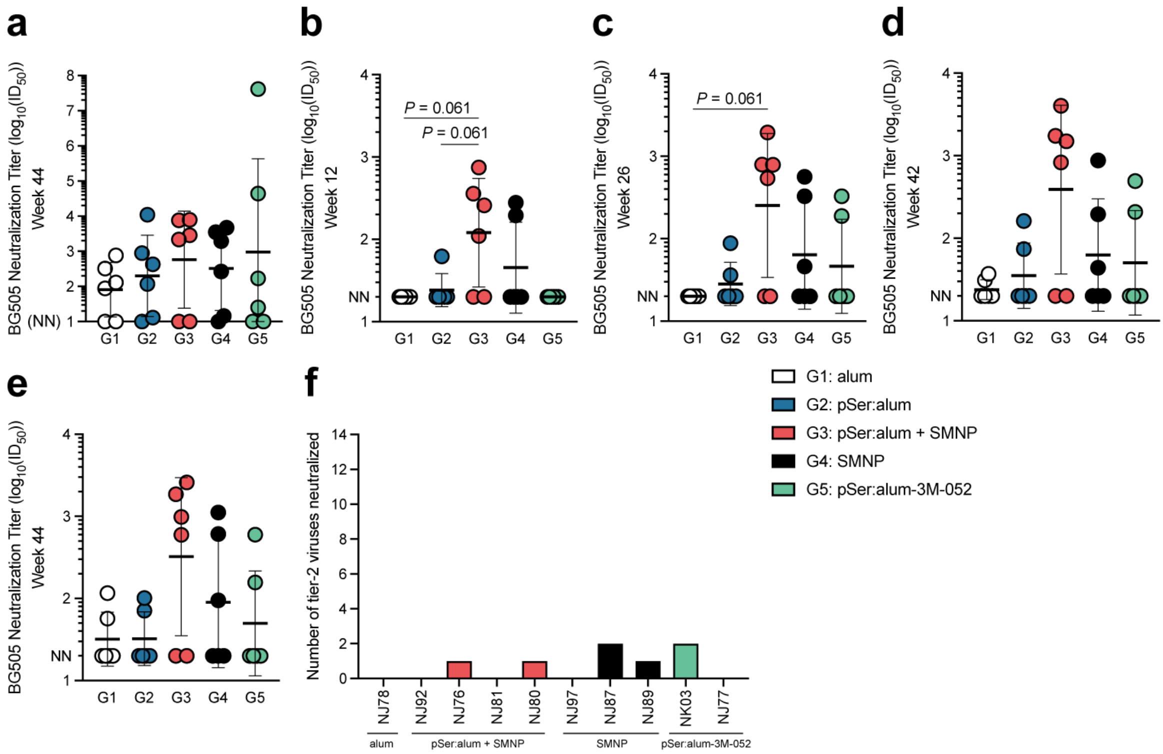

Supplementary Fig. 5 | BG505 tier-2 neutralization titers.

a, BG505 pseudovirus autologous neutralization titers at week 44. **b,** BG505 pseudovirus autologous neutralization titers at week 12, performed independently at Duke University.**c,** BG505 pseudovirus autologous neutralization titers at week 26, performed independently at Duke University. **d,** BG505 pseudovirus autologous neutralization titers at week 42, performed independently at Duke University. **e,** BG505 pseudovirus autologous neutralization titers at week 44, performed independently at Duke University. **f,** Number of heterologous tier-2 viruses neutralized (ID_{50} > 50) in the 13-virus global panel at week 42. NN = non-neutralizing. Neutralization data (Supplementary Fig. 4b) for Group 1, alum, have been previously published[1]. Mean and SEM or geometric mean and geometric SD are plotted depending on the scale in all figures unless otherwise stated. For Supplementary Fig. 5, n=6 samples per group. For 5a, samples were run in two independent experiments and values were averaged. Statistical significance was tested using either unpaired two-tailed Mann-Whitney tests or Kruskal-Wallis test with Dunn's multiple comparisons test, depending on the objectives of the study. *p < 0.05, **p < 0.01, ***p < 0.001, ****p < 0.0001. Source data are provided as a Source Data file.

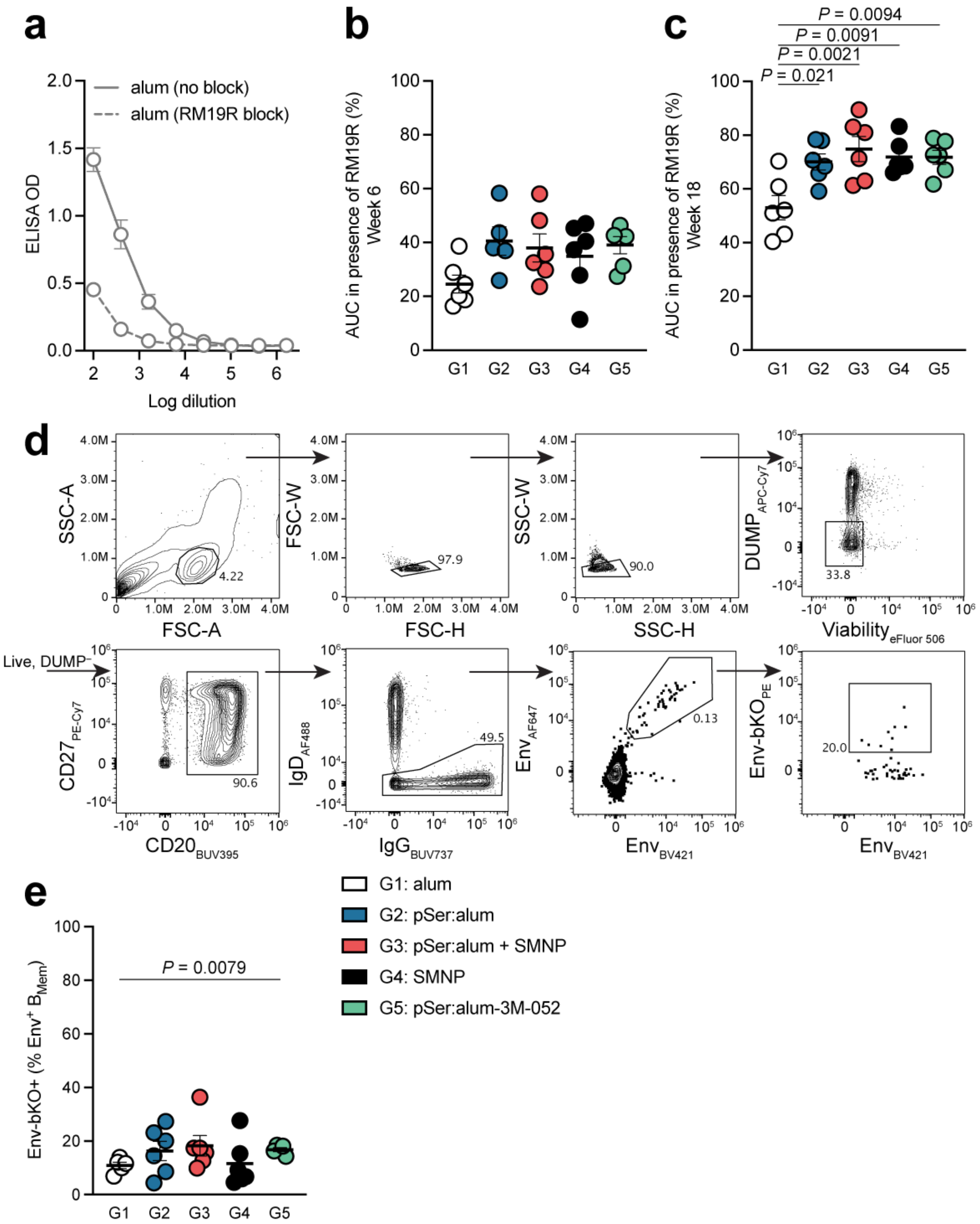

a
alum (no block)
alum (RM19R block)
ELISA OD
Log dilution
b
AUC in presence of RM19R (%) Week 6
G1 G2 G3 G4 G5
c
AUC in presence of RM19R (%) Week 18
P = 0.0094
P = 0.0091
P = 0.0021
P = 0.021
d
SSC-A
FSC-A
4.22
FSC-W
FSC-H
97.9
SSC-W
SSC-H
90.0
DUMP APC-Cy7
Viability eFluor 506
33.8
Live, DUMP−
CD27 PE-Cy7
CD20 BUV395
90.6
IgD AF488
IgG BUV737
49.5
Env AF647
Env BV421
0.13
Env-bKO PE
20.0
e
Env-bKO+ (% Env+ BMem)
P = 0.0079
G1: alum
G2: pSer:alum
G3: pSer:alum + SMNP
G4: SMNP
G5: pSer:alum-3M-052

Supplementary Fig. 6 | Non-base binding responses elicited by the different adjuvants.
a, Example curve from a competition ELISA assay with 19R, a high affinity base-specific antibody. **b,** Off-target antibody responses to the base of the trimer from serum at week 6. **c,** Off-target antibody responses to the base of the trimer from serum at week 18. **d,** Flow cytometry gating of Env-specific and non-base-binding Env-specific B_{Mem} cells. **e,** Non-base binding (Env-bKO^{+}) frequency as a percentage of Env^{+} B_{Mem} cells. For Supplementary Fig. 6, n=6 samples per group. Statistical significance for cross-competition ELISAs (a-c) was tested using Ordinary one-way ANOVA with Tukey's multiple comparisons test. Statistical significance for B_{Mem} cell frequency (d-e) was tested using either unpaired two-tailed Mann-Whitney tests or Kruskal-Wallis test with Dunn's multiple comparisons test, depending on the objectives of the study. *p < 0.05, **p < 0.01, ***p < 0.001, ****p < 0.0001. Source data are provided as a Source Data file.

a

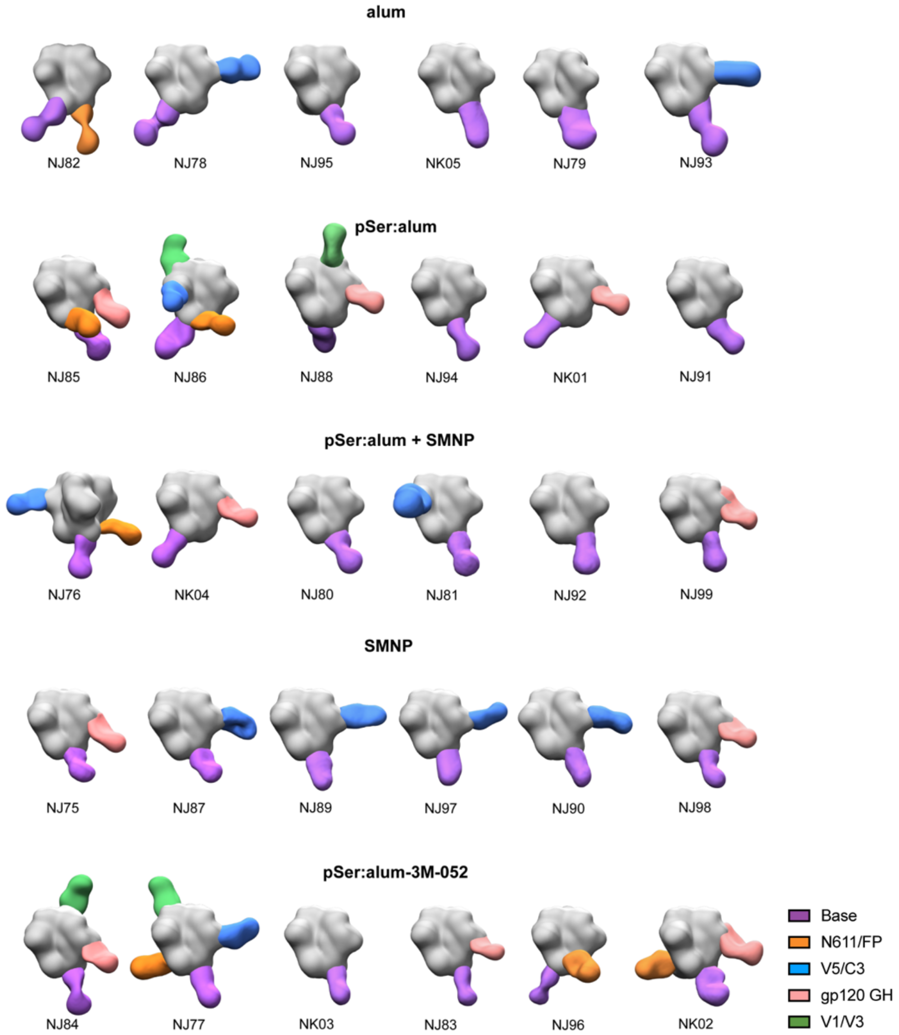

b

EMDB code	Group	Animal ID	Timepoint	Map	Polyclonal Ab epitopes
EMD-40242	2	Rh.NJ85	wk12	Main map	gp120GH
				Additional map 1	N611/FP
				Additional map 2	Base
EMD-40243	2	Rh.NJ86	Wk12	Main map	C3V5, base
				Additional map 1	N611/FP
				Additional map 2	V1V3
EMD-40244	3	Rh.NJ76	Wk12	Main map	C3V5, base
				Additional map 1	N611/FP, base
EMD-40252	3	Rh.NK04	Wk12	Main map	gp120GH, base
EMD-40254	4	Rh.NJ75	Wk12	Main map	gp120GH, base
EMD-40255	4	Rh.NJ87	Wk12	Main map	C3V5, base
EMD-40256	5	Rh.NJ84	Wk12	Main map	V1V3
				Additional map 1	gp120GH, base
EMD-40257	5	Rh.NJ77	Wk12	Main map	base
				Additional map 1	N611/FP
				Additional map 2	V1V3, base
				Additional map 3	C3V5

Supplementary Fig. 7 | Electron microscopy polyclonal epitope mapping of Week 12 responses. **a,** Composite maps of polyclonal antibody specificities against BG505 MD39 SOSIP by experimental group. EMPEM data from Group 1, alum, have been previously published[1]. **b,** Representative maps have been deposited to the EMDB under the accession codes listed in the table. EMPEM maps for Rh.NJ78 and Rh.NJ82 have been previously deposited under accession codes EMD-27609 and EMD-27610, respectively.

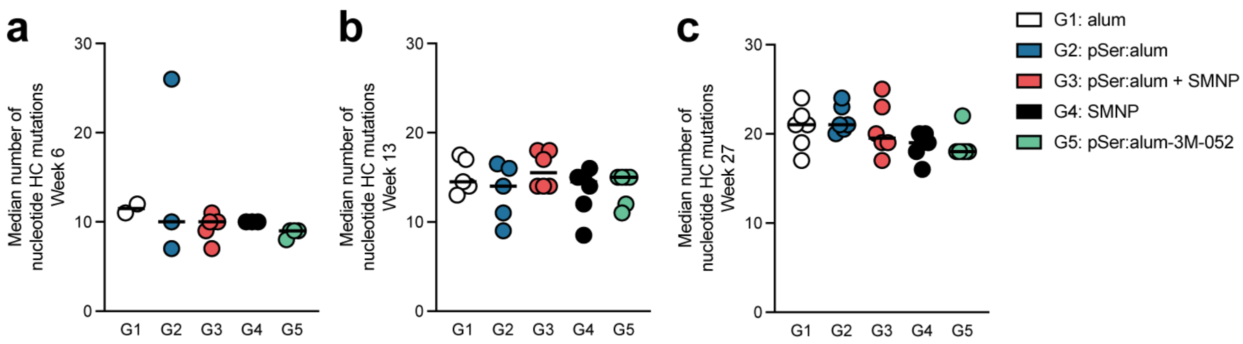

Supplementary Fig. 8 | Somatic mutation analysis of Env-binding B cells by animal.

a, The median number of nucleotide HC mutations per group at week 6. **b,** The median number of nucleotide HC mutations per group at week 13. **c,** The median number of nucleotide HC mutations per group at week 27. Where individual samples are plotted (a-c), a sample is excluded if fewer than 10 sequences were recovered. Median values are plotted. For Supplementary Fig. 8, n=12 samples (left and right LN), 6 animals per group. Source data are provided as a Source Data file.

Supplementary Table 1: Flow cytometry panel of NHP LN FNA staining for Env-specific B_{GC} cells

Antibodies	Clone	Source	Catalog #	Dilution
Alexa Fluor 647 Streptavidin	-	Invitrogen	S32357	-
BV421 Streptavidin	-	BioLegend	405225	-
LIVE/DEAD Fixable Aqua	-	Invitrogen	L34957	1:1000
Mouse anti-human CD3 BV786	SP34-2	BD Biosciences	563918	1:67
Mouse anti-human CD4 BV650	OKT4	BioLegend	317436	1:100
Mouse anti-human CD8a APC-eFluor780	RPA-T8	Thermo Fisher Scientific	47-0088-42	1:200
Mouse anti-human CD16 APC-Cy7	3G8	BioLegend	302018	1:100
Mouse anti-human CD20 Alexa Fluor 488	2H7	BioLegend	302316	1:50
Mouse anti-human CD38 PE	OKT10	NHP Reagents	PR-3802	1:20
Mouse anti-NHP CD45 BUV395	D058-1283	BD Biosciences	564099	1:100
Mouse anti-human CD71 PE-CF594	L01.1	BD Biosciences	Custom conjugate	1:20
Mouse anti-human PD-1 BV605	EH12.2H7	BioLegend	329924	1:20
Mouse anti-human CXCR5 PE-Cy7	MU5UBEE	Thermo Fisher Scientific	25-9185-42	1:20
Mouse anti-human IgG Alexa Fluor 700	G18-145	BD Biosciences	561296	1:40
Mouse anti-human IgM PerCP-Cy5.5	G20-127	BD Biosciences	561285	1:40

Supplementary Table 2: Flow cytometry panel of PBMC staining for Env-specific B_{Mem} cells

Antibodies	Clone	Source	Catalog #	Dilution
Alexa Fluor 647 Streptavidin	-	Invitrogen	S32357	-
BV421 Streptavidin	-	BioLegend	405225	-
PE Streptavidin	-	Invitrogen	S866	-
eBioscience Fixable Viability Dye eFluor 506	-	Invitrogen	65-0866-14	1:500
Mouse anti-human CD3 APC-Cy7	SP34-2	BD Biosciences	557757	1:100
Mouse anti-human CD14 APC-Cy7	M5E2	BioLegend	301820	1:100
Mouse anti-human CD16 APC-eFluor780	eBioCB16	Thermo Fisher Scientific	47-0168-42	1:100
Mouse anti-human CD20 BUV395	2H7	BD Biosciences	563781	1:100
Mouse anti-human CD27 PE-Cy7	O323	BioLegend	302838	1:50
Goat anti-human IgD Ax488	Polyclonal	Southern Biotech	2030-30	1:50
Mouse anti-human IgG BUV737	G18-145	BD Biosciences	612819	1:100
Mouse anti-human IgM BV605	G20-127	BD Biosciences	562977	1:50

Supplementary Table 3: Flow cytometry AIM and ICS panel staining for Env-specific T cells

Antibodies	Clone	Source	Catalog #	Dilution
LIVE/DEAD Fixable Blue	-	Invitrogen	L23105	
GolgiPlug	-	BD Biosciences	555029	-
GolgiStop	-	BD Biosciences	554724	-
Mouse anti-human CD40	HB14	Miltenyi	130-094-133	1:200
Mouse anti-human CXCR5 PE-Cy7	MU5UBEE	Thermo Fisher Scientific	25-9185-42	1:100
Mouse anti-human CCR7 BV650	G043H7	BioLegend	353233	1:100
Mouse anti-human CD69 PE-Cy5	FN50	BioLegend	310908	1:250
Mouse anti-human CD137 (4-1BB) BV421	4B4-1	BioLegend	309819	1:250
Mouse anti-human CD25 BV605	BC96	BioLegend	302631	1:250
Mouse anti-human CD40L BB515	24-31	BD Biosciences	568170	1:250
Mouse anti-human CD134 (OX40) PE	L106	BD Biosciences	340420	1:250
Mouse anti-human CD8 BUV496	RPA-T8	BD Biosciences	612943	1:100
Mouse anti-human CD14 APC-Cy7	M5E2	BioLegend	301820	1:100
Mouse anti-human CD16 APC-eFluor780	eBioCB16	Thermo Fisher Scientific	47-0168-42	1:100
Mouse anti-human CD20 APC-Cy7	2H7	BioLegend	302314	1:100
Mouse anti-human CD3 BUV395	SP34-2	BD Biosciences	564117	1:100
Mouse anti-human CD4 PerCP-Cy5.5	OKT4	BioLegend	317428	1:100
Mouse anti-human PD-1 BV785	EH12.2H7	BioLegend	329929	1:100
Mouse anti-human CD45RA PE-CF594	5H9	BD Biosciences	565419	1:100
Armenian Hamster anti-ICOS BV480	C398.4A	BD Biosciences	566087	1:100
Mouse anti-human IFN-γ BUV737	4S.B3	BD Biosciences	612845	1:100

Rat anti-human IL-2 BV750	MQ1-17H12	BD Biosciences	566361	1:200
Mouse anti-human TNF-α BV711	MAb11	BioLegend	502940	1:200
Mouse anti-human Granzyme B Alexa Fluor 700	GB11	BD Biosciences	560213	1:1000
Mouse anti-human IL-21 Alexa Fluor 647	3A3-N2.1	BD Biosciences	560493	1:200
Human Fc Block	Fc1	BD Biosciences	564220	1:20

Supplementary References

1. Lee, J. H. *et al.* Long-primed germinal centres with enduring affinity maturation and clonal migration. *Nature* 1–7 (2022) doi:10.1038/s41586-022-05216-9.
