# Supplementary material for: A combined adjuvant approach primes robust germinal center responses and humoral immunity in non-human primates
Source: Nat Commun. 2023 Nov 4;14:7107. doi: 10.1038/s41467-023-42923-x (PMC10625619; doi:10.1038/s41467-023-42923-x)
Supplement: Supplementary file 3 — Reporting Summary [file 41467_2023_42923_MOESM3_ESM.pdf]

Corresponding author(s): Shane Crotty, Darrell Irvine

Last updated by author(s): Sep 20, 2023

Reporting Summary

Nature Portfolio wishes to improve the reproducibility of the work that we publish. This form provides structure for consistency and transparency in reporting. For further information on Nature Portfolio policies, see our Editorial Policies and the Editorial Policy Checklist.

Statistics

For all statistical analyses, confirm that the following items are present in the figure legend, table legend, main text, or Methods section.

n/a	Confirmed	
☐	☒	The exact sample size (*n*) for each experimental group/condition, given as a discrete number and unit of measurement
☐	☒	A statement on whether measurements were taken from distinct samples or whether the same sample was measured repeatedly
☐	☒	The statistical test(s) used AND whether they are one- or two-sided<br>*Only common tests should be described solely by name; describe more complex techniques in the Methods section.*
☐	☒	A description of all covariates tested
☐	☒	A description of any assumptions or corrections, such as tests of normality and adjustment for multiple comparisons
☐	☒	A full description of the statistical parameters including central tendency (e.g. means) or other basic estimates (e.g. regression coefficient) AND variation (e.g. standard deviation) or associated estimates of uncertainty (e.g. confidence intervals)
☐	☒	For null hypothesis testing, the test statistic (e.g. *F*, *t*, *r*) with confidence intervals, effect sizes, degrees of freedom and *P* value noted<br>*Give P values as exact values whenever suitable.*
☒	☐	For Bayesian analysis, information on the choice of priors and Markov chain Monte Carlo settings
☒	☐	For hierarchical and complex designs, identification of the appropriate level for tests and full reporting of outcomes
☒	☐	Estimates of effect sizes (e.g. Cohen's *d*, Pearson's *r*), indicating how they were calculated

Our web collection on statistics for biologists contains articles on many of the points above.

Software and code

Policy information about availability of computer code

Data collection	Leginon was used to automate EM data collection
Data analysis	All data were processed with previously published software and code. Flow cytometry data were analyzed in FlowJo v10 (BD Biosciences). ELISpot, ELISAs and neutralization data were analyzed in Prism 9 (GraphPad). Sequencing data were compiled and processed using CellRanger v6.1.2 (10X Genomics) and hashing antibodies were demultiplexed by Seurat v4. BCR data were analyzed using packages (Change-O v1.3.0; SHazaM v1.1.2; Alakazam v1.2.1) available from the Immcantation Portal v4.4.0 and VSEARCH v2.21.1. Statistics were calculated in Prism 9 (GraphPad). EM data were processed using Appion and Relion 3.0. EM data were assessed with UCSF Chimera 1.13, which was also used to generate figures.

For manuscripts utilizing custom algorithms or software that are central to the research but not yet described in published literature, software must be made available to editors and reviewers. We strongly encourage code deposition in a community repository (e.g. GitHub). See the Nature Portfolio guidelines for submitting code & software for further information.

Data

Policy information about availability of data

All manuscripts must include a data availability statement. This statement should provide the following information, where applicable:

- Accession codes, unique identifiers, or web links for publicly available datasets
- A description of any restrictions on data availability
- For clinical datasets or third party data, please ensure that the statement adheres to our policy

The BCR sequencing data generated in this study are available in the Sequence Read Archive (SRA) under accession code PRJNA1016452 [https://www.ncbi.nlm.nih.gov/sra/?term=PRJNA1016452]. The 3D EM reconstructions are available from the Electron Microscopy Bank under the following EMDB codes: EMD-40242 [https://www.ebi.ac.uk/emdb/EMD-40242], EMD-40243 [https://www.ebi.ac.uk/emdb/EMD-40243], EMD-40244 [https://www.ebi.ac.uk/emdb/EMD-40244], EMD-40252 [https://www.ebi.ac.uk/emdb/EMD-40252], EMD-40254 [https://www.ebi.ac.uk/emdb/EMD-40254], EMD-40255 [https://www.ebi.ac.uk/emdb/EMD-40255], EMD-40256 [https://www.ebi.ac.uk/emdb/EMD-40256], and EMD-40257 [https://www.ebi.ac.uk/emdb/EMD-40257]. Sequencing data and electron microscopy particle stacks are also available upon request. Source data are provided with this paper.

Research involving human participants, their data, or biological material

Policy information about studies with human participants or human data. See also policy information about sex, gender (identity/presentation), and sexual orientation and race, ethnicity and racism.

Reporting on sex and gender	Not applicable
Reporting on race, ethnicity, or other socially relevant groupings	Not applicable
Population characteristics	Not applicable
Recruitment	Not applicable
Ethics oversight	Not applicable

Note that full information on the approval of the study protocol must also be provided in the manuscript.

Field-specific reporting

Please select the one below that is the best fit for your research. If you are not sure, read the appropriate sections before making your selection.

[x] Life sciences [ ] Behavioural & social sciences [ ] Ecological, evolutionary & environmental sciences

For a reference copy of the document with all sections, see nature.com/documents/nr-reporting-summary-flat.pdf

Life sciences study design

All studies must disclose on these points even when the disclosure is negative.

Sample size	6 rhesus macaques were assigned per immunization group. A power analysis was previously performed to determine the optimal sample size to distinguish meaningful statistical differences in nAb titers between animal groups, based on previous studies (Pauthner, et al., Immunity 2017). Where available, data points from left and right lymph nodes from each animal were considered as two separate data points.
Data exclusions	Where there were little or no cells in some of the LN FNA samples, data were excluded from assessment by flow cytometry. For bulk GC data inclusion in the LN FNA samples, a threshold of 250 total B cells in the sample was used. For Env-specific BGC cell data inclusion, a threshold of 75 total BGC cells was used. For memory B cell analysis , a threshold of 10 Env+ Bmem cells were used for inclusion. PBMCs from NK03 from Group 5, pSer:alum-3M-052, was not collected and therefore not included in BMem cell analysis. For the T cell analysis, Sample NK04 from Group 3, pSer:alum + SMNP, was excluded from antigen-specific T cell analysis at week 2 and week 10 due to the viability of the cells. For somatic hypermutation analysis, when data was graphed on an individual animal basis, a minimum of 10 sequences recovered was required to properly calculate medians. For diversity analysis, a minimum of 50 sequences recovered was required to better represent the diversity of the sequences elicited from immunization.
Replication	Rhesus macaque immunizations were not repeated due to difficulty in having access to a large number of animals per immunization group. Neutralization assays were repeated at twice at Scripps, with the data averaged between replications or once at Duke. Most replications were successful, some samples were excluded at week 42 due to sample availability (there were not enough sample volume to run a repeat experiment). Some neutralization assays were also performed independently by two distinct laboratories to confirm reproducibility. Bone marrow ELISpots were successfully performed twice. Other experiments such as flow cytometry using LN FNA samples and PBMCs (BGC, GC-TFH, AIM assays, BMem analysis), EMPEM, ELISAs (duplicate wells) were performed once.
Randomization	All experiments were performed using samples from rhesus macaques from one study. These rhesus macaques were weight, age, sex-matched, then randomly divided such that there was an even number of female to male macaques in each group.
Blinding	Investigators were not blinded. We performed only quantitative measurements to compare immune responses between multiple adjuvants.

We thought it was OK to keep the study unblinded as there were no measurements that would have bias (like scoring disease severity, etc.).

Reporting for specific materials, systems and methods

We require information from authors about some types of materials, experimental systems and methods used in many studies. Here, indicate whether each material, system or method listed is relevant to your study. If you are not sure if a list item applies to your research, read the appropriate section before selecting a response.

Materials & experimental systems

n/a	Involved in the study
☐	☒ Antibodies
☐	☒ Eukaryotic cell lines
☒	☐ Palaeontology and archaeology
☐	☒ Animals and other organisms
☒	☐ Clinical data
☒	☐ Dual use research of concern
☒	☐ Plants

Methods

n/a	Involved in the study
☒	☐ ChIP-seq
☐	☒ Flow cytometry
☒	☐ MRI-based neuroimaging

Antibodies

Antibodies used

Alexa Fluor 647 streptavidin (Invitrogen, Cat #S32357), BV421 streptavidin (BioLegend, Cat #405225), PE streptavidin (Invitrogen, Cat #S866), eBioscience Fixable Viability Dye eFluor 506 (Invitrogen, Cat #65-0866-14, 1:500), LIVE/DEAD Fixable Aqua (Invitrogen, Cat #L34957, 1:1000), mouse anti-human CD3 BV786 (SP34-2, BD Biosciences, Cat #563918, 1:67), mouse anti-human CD3 APC-Cy7 (SP34-2, BD Biosciences, Cat #557757, 1:100), mouse anti-human CD4 BV650 (OKT4, BioLegend, Cat #317436, 1:100), mouse anti-human CD8a APC-eFluor 780 (RPA-T8, Thermo Fisher Scientific, Cat #47-0088-42, 1:200), mouse anti-human CD14 APC-Cy7 (M5E2, BioLegend, Cat #301820, 1:100), mouse anti-human CD16 APC-eFluor 780 (eBioCB16, Thermo Fisher Scientific, Cat #47-0168-42, 1:100), mouse anti-human CD16 APC-Cy7 (3G8, BioLegend, Cat #302018, 1:100), mouse anti-human CD20 Alexa Fluor 488 (2H7, BioLegend, Cat #302316, 1:50), mouse anti-human CD20 BUV395 (2H7, BD Biosciences, Cat #563781, 1:100), mouse anti-human CD27 PE-Cy7 (O323, BioLegend, Cat #302838, 1:50), mouse anti-human CD38 PE (OKT10, NHP Reagents, Cat #PR-3802, 1:20), mouse anti-NHP CD45 BUV395 (D058-1283, BD Biosciences, Cat #564099, 1:100), mouse anti-human CD71 PE-CF594 (L01.1, BD Biosciences, Custom conjugate, 1:20), mouse anti-human PD-1 BV605 (EH12.2H7, BioLegend, Cat #329924, 1:20), mouse anti-human CXCR5 PE-Cy7 (MU5UBEE, Thermo Fisher Scientific, Cat #25-9185-42, 1:20-1:100), goat anti-human IgD Alexa Fluor 488 (polyclonal, Southern Biotech, Cat #2030-30, 1:50), mouse anti-human IgG Alexa Fluor 700 (G18-135, BD Biosciences, Cat #561296, 1:40), mouse anti-human IgG BUV737 (G18-145, BD Biosciences, Cat #612819, 1:100), mouse anti-human IgM PerCP-Cy5.5 (G20-127, BD Biosciences, Cat #561285, 1:40), mouse anti-human IgM BV605 (G20-127, BD Biosciences, Cat #562977, 1:50), TotalSeq-C anti-human Hashtag antibody 1-10 (LNH-94 and 2M2, Biolegend, Cat #394661, #394663, #394665, #394667, #394669, #394671, #394673, #394675, #394677, #394679, 4μL), LIVE/DEAD Fixable Blue (Invitrogen, Cat #L23105), GolgiPlug (BD Biosciences, Cat #555029), GolgiStop (BD Biosciences, Cat #554724), mouse anti-human CD40 (HB14, Miltenyi, Cat #130-094-133, 1:200), mouse anti-human CCR7 BV650 (G043H7, BioLegend, Cat #353233, 1:100), mouse anti-human CD69 PE-Cy5 (FN50, BioLegend, Cat #310908, 1:250), mouse anti-human CD137 (4-1BB) BV421 (4B4-1, BioLegend, Cat #309819, 1:250), mouse anti-human CD25 BV605 (BV96, BioLegend, Cat #302631, 1:250), mouse anti-human CD40L BB515 (24-31, BD Biosciences, Cat #568170, 1:250), mouse anti-human CD134 (OX40) PE (L106, BD Biosciences, Cat #340420, 1:250), mouse anti-human CD8 BUV496 (RPA-T8, BD Biosciences, Cat #612943, 1:100), mouse anti-human CD20 APC-Cy7 (2H7, BioLegend, Cat #302314, 1:100), mouse anti-human CD3 BUV395 (SP34-2, BD Biosciences, Cat #564117, 1:100), mouse anti-human CD4 PerCP-Cy5.5 (OKT-4, BioLegend, Cat #317428, 1:100), mouse anti-human PD-1 BV785 (EH12.2H7, BioLegend, Cat #329929, 1:100), mouse anti-human CD45RA PE-CF594 (5H9, BD Biosciences, Cat #565419, 1:100), Armenian Hamster anti-ICOS BV480 (C398.4A, BD Biosciences, Cat #566087, 1:100), mouse anti-human IFN-γ BUV737 (4S.B3, BD Biosciences, Cat #612845, 1:100), rat anti-human IL-2 BV750 (MQ1-17H12, BD Biosciences, Cat #566361, 1:200), mouse anti-human TNF-α BV711 (Mab11, BioLegend, Cat #502940, 1:200), mouse anti-human Granzyme B Alexa Fluor 700 (GB11, BD Biosciences, Cat #560213, 1:1000), mouse anti-human IL-21 Alexa Fluor 647 (3A3-N2.1, BD Biosciences, Cat #560493, 1:200), and human Fc block (Fc1, BD Biosciences, Cat #564220, 1:20).

Validation

Validation can be found in the respective manufacturer website or our previous NHP studies (Cirelli, et al., Cell 2019, Pauthner et al., Immunity 2017, Reiss, et al., PLOS One 2017, Lee, et al., Nature 2022).

Eukaryotic cell lines

Policy information about cell lines and Sex and Gender in Research

Cell line source(s)	Human (HEK293F) from commercial supplier (ThermoFisher Scientific)
Authentication	The HEK293F cell line is authenticated by the commercial supplier (ThermoFisher)
Mycoplasma contamination	Cell lines were not tested for Mycoplasma contamination.
Commonly misidentified lines (See ICLAC register)	No commonly misidentified cell lines were used in the study.

Animals and other research organisms

Policy information about studies involving animals; ARRIVE guidelines recommended for reporting animal research, and Sex and Gender in Research

Laboratory animals	Rhesus macaques (Macaca mulatta), aged between 3.5 to 5 years at the time of the priming immunization, were used.
Wild animals	The study did not involve wild animals.
Reporting on sex	Both females and male rhesus macaques were used, and were divided randomly between immunization groups, such that there were an even number of females and males in each group (3 females and 3 males).
Field-collected samples	The study did not involve samples collected from the field.
Ethics oversight	The study was approved by the Tulane University Institutional Animal Care and Use Committee (IACUC).

Note that full information on the approval of the study protocol must also be provided in the manuscript.

Flow Cytometry

Plots

Confirm that:

- [x] The axis labels state the marker and fluorochrome used (e.g. CD4-FITC).
- [x] The axis scales are clearly visible. Include numbers along axes only for bottom left plot of group (a 'group' is an analysis of identical markers).
- [x] All plots are contour plots with outliers or pseudocolor plots.
- [x] A numerical value for number of cells or percentage (with statistics) is provided.

Methodology

Sample preparation	Lymph node fine needle aspirates (LN FNAs) were performed by a veterinarian to sample the left and right draining inguinal LNs (iLNs), which were identified by palpation. The biopsy site was aseptically prepared and a 22-gauge 1.5-inch needle attached to a 3-mL syringe was passed into the LN 4-5 times. Samples were placed into RPMI containing 10% fetal bovine serum (FBS) and 1X penicillin/streptomycin (pen/strep). Ammonium-Chloride-Potassium (ACK) lysing buffer was used if the sample was contaminated with red blood cells. LN FNA cells were counted and divided between different assays; any extra cells were frozen down and stored in liquid nitrogen until further analysis. Cells were incubated with the WT MD39 probes for 30 minutes at 4ºC and then with surface antibodies for an additional 30 minutes at 4ºC. Where MD39-bKO probes were used, cells were first incubated with MD39-bKO probes for 20 minutes at 4ºC, then WT MD39 probes for 30 minutes at 4ºC, and finally with the surface antibodies for 30 minutes at 4ºC, similar to previously described protocols12,20. For samples being sorted, anti-human TotalSeq-C hashtag antibodies (BioLegend) were added to each individual sample at a concentration of 2 µg per 5 million cells along with the surface antibody master mix.
Instrument	All samples were either acquired on a FACSAria Fusion (BD Biosciences), a LSRFortessa (BD Biosciences), or a Cytek Aurora (Cytek Biosciences), depending on the experiment, or sorted on a FACSAria Fusion (BD Biosciences).
Software	BD FACSDiva was used for data acquisition from all BD machines. SpectroFlo was used to acquire data from the Cytek Aurora. FlowJo v10 was used for all data analysis.
Cell population abundance	Information regarding cell abundance can be found in Fig. 1c, e; Fig. 2; Fig. 5e-g; Supplementary Fig. 2; Supplementary Fig. 3; Supplementary Fig. 6e, where GC B cells, Memory B cells, Env-specific T cells were all graphed.
Gating strategy	All complete gating strategies can be found in the Supplementary Figures (Supplementary Fig. 2a, 3c, 6d).

- [x] Tick this box to confirm that a figure exemplifying the gating strategy is provided in the Supplementary Information.
